# Population Health Challenges and Evaluative Thinking: Rapid Responses in the Time of COVID-19

**DOI:** 10.1177/1035719X231175884

**Published:** 2023-05-16

**Authors:** Katina D’Onise, Katherine Pontifex

**Affiliations:** 1066 Wellbeing SA, Adelaide, SA, Australia

**Keywords:** developmental evaluation, population health, COVID-19, evaluation, Lean

## Abstract

There are multiple complex challenges that the population health sector faces to improve the health of the community, and the sector must work efficiently and effectively to make the most of the committed funds, sometimes in contexts of uncertainty. This article explores, through the case study of the South Australian COVID-19 Vaccine Hesitancy Project, the application of pillars from the Lean Start-up model of setting a clear vision, and creation of a series of minimum viable products to enable work with the community to commence as quickly as possible. This article draws parallels between this corporate model and the notion of evaluative thinking as well as adaptive evaluation approaches, demonstrates the interest from population health program and policy actors in adaptive practices and proposes a continuing opportunity for the evaluation profession in this space.

**What we already know:**

• Evaluative thinking, alongside the technical knowledge and skills of evaluation practice, has been proposed as an essential element for achieving quality evaluation practice (Buckley et al., 2015).
• Formative and summative evaluation approaches have been complemented in recent years by adaptive evaluation approaches applied in dynamic contexts (Patton, 2011).

**The original contribution the article makes to theory and/or practice:**

• This article explores what population health can learn from the Lean Start-up model used in the corporate world and the parallels with the overall notion of evaluative thinking and with adaptive evaluation approaches; specifically developmental evaluation.
• This case study provides an example of our program and policy colleagues seeking ways to address complex issues or respond in dynamic contexts. It indicates the importance of the evaluation profession continuing to promote evaluative thinking and adaptive evaluation approaches as part of our toolbox.

## Introduction

The population health sector faces major challenges. People live, in many instances, in fundamentally unhealthy environments where they are surrounded by social, cultural and physical spaces that are not health promoting and lead to poor health outcomes (Irwin et al., 2006). Compounding these challenges, the public health budget is competing with the very much larger pool of funds of corporations that seek to shift our choices in an unhealthy direction, and the population health agenda is fundamentally competing with a range of other more pressing concerns for the community, such as housing and food affordability (Kickbush et al., 2016; Australian Bureau of Statistics, 2022a). To meet these demands, population health must work at a system level to bring about changes to improve health outcomes with a high enough dose and in a way that is sustainable over time. Population health must work more efficiently and effectively within the available public health budget for sustainable health gains.

The COVID-19 pandemic presented many challenges but sometimes also opportunities for the community and the population health sector (Australian Bureau of Statistics, 2022b; Chiraboga et al., 2020). The South Australian COVID-19 Vaccine Hesitancy Project was implemented across 2021–2022 in a dynamic context and was viewed as an opportunity to use processes and techniques from the Lean Start-Up model (usually seen in the corporate sector) to explore what value the model might contribute to population health outcomes.

At the conclusion of the project, the population health and evaluation actors reflected on the parallels of this model with evaluative thinking and with adaptive evaluation approaches and these are addressed in this article.

## The Lean Start-up Model

The Lean Start-up model has been around for over a decade in the start-up world, and over this time has become a well-known and frequently applied approach (Ries, 2011). At the heart of the approach is an understanding that a start-up is attempting to bring forth a new product or service that is successful in the context of extreme uncertainty. While there has been limited systematic research on the benefits of the Lean Start-up model, there have been a few studies, the most recent of which found evidence of components of the model being key to a successful business (Welter et al., 2021; Ghezzi, 2019). Many terms that come from the Lean Start-up model have become commonplace, such as ‘failing fast’, ‘pivot’ and ‘minimum viable product’.

Lean Start-up evolved from taking the principles of lean manufacturing, applying the concepts to make a framework for entrepreneurship or innovation. The model favours experimentation over elaborate planning, customer feedback over intuition and iterative design over traditional ‘big design up front’ development (Blank, 2013). It consists of three major components (Ries, 2011) summarised below.

Pillar one of the model relates to the vision of the enterprise. Each start-up has a vision that is generally set, with a product (or service) and a strategy to get to the vision – the latter two can and do change in the process of reaching the vision. There is an emphasis placed on the importance of learning as we go from interactions with real customers about what would and would not work and adapting the product to better suit. The learning process is characterised as relating to repeated experiments, specifically designed to understand the effectiveness of each component of the product being offered through testing the assumptions made about the product. For example, a value hypothesis regarding whether a product delivers value to customers is tested through a small experiment. This process of experimentation focuses on testing how people actually behave, rather than how they report they would behave.

The second pillar delves more deeply into the process of learning and adapting the product to better suit the customer, known as the build-measure-learn feedback loop. This is a deliberate strategy of learning directly from the customer about how they find the product, using both quantitative and qualitative information. The model proposes that it is important to get feedback early in the process of product development as the product then can be tailored to the customer as quickly as possible, allowing the most rapid path to an effective product. This early model is known as a minimum viable product, with features of what is wanted in the product, but it is not fully developed. There is a deliberate strategy to identify and collect actionable metrics in the product feedback rather than nice to collect or ‘vanity metrics’ (Rogers, 2018). Another way to think about vanity metrics are those that might be easy to collect (for example, hits on a website) but that do not provide information about the hypothesis being tested or cannot be used directly to improve the product being provided. The assumptions inbuilt into the minimum viable product are systematically tested, measuring the baseline state in addition to the actionable metrics defined in this stage. At this stage, early adopters can provide critical information to help shape the product going forward. There is also consideration to how the product performs within different cohorts of people, and this information is also collected for the process of refinement of the product.

The third pillar relates to the process of ensuring scale or growth and an ongoing process of adapting to the information which is continuously collected to refine and enhance the product. In this stage, the start-up identifies the specific engine of growth (or the way in which growth is driven) that will be employed and then directs energy to that engine, including metrics to understand performance. Engines of growth can fall into three categories: paid (higher revenue and/or reduced cost), viral (existing customers bring new customers) or sticky (high customer retention rate). This stage also involves identifying how to structure business operations to support lean thinking in the long term, to enable an ongoing culture of innovation and avoid losing the agile edge. This can, for example, include incubating innovation teams within the organisation.

## The Lean Start-up and Population Health

Why would population health want to consider using a corporate sector model? The many challenges faced by population health require the exploration of new ways to achieve outcomes, and parallels between the population health context and the corporate context were identified. The basic tenet of building a product that will be tightly tailored to the needs of the customer so that it is embraced by the customer within a complex world can be considered to be akin to the need to promote behaviour change to improve health and wellbeing. A minimum viable product as a product (more commonly a service or system change in population health) that is able to be tested in the real world using actionable metrics and a deliberate strategy of testing assumptions has the potential to generate outcomes on a more rapid timeframe than we would typically be able to achieve using more traditional methods of lengthy planning of a complete product which we then use to bring about change. Thinking about cohorts of people in the Lean Start-up context is akin to thinking about the different communities that make up our society and tailoring to suit the community’s needs. Thinking about seeking meaningful feedback, both qualitative and quantitative on a product could be considered akin to community consultation, although in public health the preference is in many instances to extend this into community engagement (World Health Organization, 2020).

While there are a number of parallels, there is also some divergence. This can be seen particularly in the pillar three stage of scale and ongoing adaptation. Population health is also concerned about the need for scale; however, the *approach* diverges. For example, there are a range of features of a population health approach which support sustainability that use entirely different mechanisms than market forces such as legislation, policy or ongoing budgetary allocation by government as a sign of success in sustainability. Community engagement in population health goes beyond gathering information about services to be offered to the community, to involve community driven services or interventions, designed in partnership with the community. Additionally, behaviour change at the population level for health requires a multitude of decisions on a daily basis, whereas purchasing a product requires far fewer decision points. The systems required to support behaviour change for health therefore are more complex, aiming to change the environment to support healthy decision making and so involving a vast array of interventions to change the environment (World Health Organization, 1986).

## Evaluative Thinking and Adaptive Evaluation Practice

The Lean Start-up model may seem familiar to many in the evaluation profession.

Firstly, the model speaks to the overarching notion of evaluative thinking, defined by Buckley et al. (2015) as: ‘critical thinking applied in the context of evaluation, motivated by an attitude of inquisitiveness and a belief in the value of evidence, that involves identifying assumptions, posing thoughtful questions, pursuing deeper understanding through reflection and perspective taking and informing decisions in preparation for action’ (p. 4).

According to Buckley et al. (2015), evaluative *thinking*, alongside the knowledge and skills of evaluation *doing*, is an essential element for achieving high quality evaluation practice, especially when all actors involved in the evaluation embrace this notion. The Lean Start-up model applies this ‘attitude of inquisitiveness’ and ‘belief in the value of evidence’ in its processes.

In addition, there are parallels between the Lean Start-up model and evaluation practice with a learning or improvement purpose – ‘interactive’ forms of evaluation including approaches such as Action Research (Owen, 2006). These parallels are particularly notable in developmental evaluation which is in an adaptive evaluation approach which complements the ‘traditional’ focus on the improvement (formative evaluation) or judgement of the merit or worth (summative evaluation) for a defined intervention (Patton, 2011). Developmental evaluation ‘[s]upports the development of innovations and adaptation of interventions in **dynamic environments’** [emphasis added] (Patton, 2011, p. 23) which is aligned with Lean’s focus on the creation of a new product in the context of uncertainty. In addition, developmental evaluation requires social innovators to have a strong vision (Patton, 2011) (akin to the Lean ‘vision’) and includes a process of ‘gathering real-time data to inform ongoing decision making adaptations’ Patton, 2011, p. 1) which aligns with Lean’s ‘actionable metrics’. Indeed, ‘originators [of developmental evaluation] liken their approach to **the role of research &development in the private sector product development process** because **it facilitates real-time, or close to real-time, feedback to program staff thus facilitating a continuous development loop’** [emphasis added] (Better Evaluation, 2022).

## A Case Study: The South Australian COVID-19 Vaccine Hesitancy Project

To illustrate the potential value of the Lean Start-up model (and, by extension, evaluative thinking as a way of working, and adaptive evaluation as an alternative approach), an intrinsic case study of the South Australian COVID-19 Vaccine Hesitancy Project is presented (Stake, 1995). Intrinsic in this context refers to using the case study for the purpose of understanding the case itself and not for the purpose of comparison or generalisation.

This case study takes place in the context of complexity.

Wellbeing SA is responsible for the South Australian Population Health Survey which is an ongoing survey of a random sample of South Australians of all ages using a landline and mobile phone platform, with around 7000 participants a year in a typical year (Government of South Australia, 2022).

As part of the COVID-19 pandemic response in South Australia, the South Australian Population Health Survey was adapted from April 2020 to collect real-time data with a focus on syndromic surveillance for acute respiratory illness and other COVID-19 safe behaviours. In early 2021, the survey was adapted again to understand the willingness of South Australians to be vaccinated. By September 2021, it was apparent that there were a number of communities who were not being vaccinated against COVID-19 at the rate that was seen across the whole of the South Australian population. The Aboriginal community, people with a mental health diagnosis and some people from culturally and linguistically diverse (CALD) communities had a higher degree of hesitancy to be vaccinated. This differed as well by age, with parents reporting a higher degree of hesitancy for their children than was reported for adults (Wang et al., 2022). Wellbeing SA was asked to deploy an approach to increasing vaccination in these communities rapidly.

### How Did the Approach Line up With the Lean Start-Up Model and What Could be Learnt for Future Population Health Projects?

Implementation of the South Australian COVID-19 Vaccine Hesitancy Project exhibited the key elements of the first two pillars of the Lean model; identification of the vision (pillar one) and use of repeated build-measure-learn experiments using iterative minimum viable products which were tested with early adopters (pillar two). This is described in more detail below.

The vision was to encourage vaccination in the different target communities. Over time, this vision was refined to ensure everyone had access to vaccination, so they had a choice about whether to be vaccinated or not. In the phase with equivalence to the Lean Start-up pillar two build-measure-learn stage, a broad strategy was designed which, in reality, was a series of hypotheses on what it was thought would work. These hypotheses drew on the extensive and mature literature for what works to reduce vaccine hesitancy and the frameworks provided by a community development approach (Jarrett et al., 2015; Leask et al., 2021).

Each element of the strategy was ‘built’ in what Patton (2011) describes as Developmental Evaluation’s ‘*action in the muddled middle’* (p. 177): a combination of ‘*top-down change processes centred on best practice models and effective principles’* plus using ‘*local knowledge, grassroots innovation, adaptation and emergence’* (p. 180). This enabled a responsiveness to local conditions and needs.

There were a series of what could be considered minimum viable products:

• A training package was rapidly developed for health staff but also for non-health workers and the community to better understand the COVID-19 vaccines and to dispel myths about the vaccine that were circulating through the community. This included information from the literature and from the advice and guidance of vaccine experts across Australia to fast track the learning.
• A grant scheme was proposed, and all grant documents were prepared rapidly to support rapid roll out. This grant program was targeted at key under-vaccinated communities, providing funds to support community identified and led initiatives to increase COVID-19 vaccination in their community, for example, through bespoke communications, resources and community events.
• A governance structure to support this project which was brought together within days.

In testing the ‘products’, early adopters were approached: Wellbeing SA staff were the first to undergo training, followed by staff from Local Health Networks, leading to adaptation of the training package over time to different communities and also different purposes. The grant process was iteratively adapted to meet the needs more fully of the targeted communities. The governance groups’ expertise was used to help tailor the grants scheme, in addition to learning from the early applications for grant funding.

### From there, How Did the Project Learn?

The South Australian COVID-19 Vaccine Hesitancy Project had a clear advantage over many other population health or community development projects: the outcome could be measured very quickly, precisely and with limited bias, that is, vaccination. These were clear actionable metrics that were the focus of the evaluation, rather than the vanity metrics of the number of people attending training or the number of community members attending events. At the beginning of the project, there were available data for the Aboriginal community and young people, but there were no available data to understand vaccination in people from CALD backgrounds and people with a mental health diagnosis. At this stage, proxy data on willingness to vaccinate and report of vaccination from the South Australian Population Health Survey were used to guide efforts. As the project evolved, improving the quality of vaccination data for these additional two cohorts was a priority for the team in order to guide efforts for all four cohorts.

Data and understanding the vaccination rate by cohorts of people were discussed with every governance meeting, held on a weekly basis. These data, broken down into age groups, geographic areas and communities guided decision making on what to focus on next.

Monthly gatherings with the implementation team were included to discuss what was working and what was not, and how this differed by cohort. Non-effective strategies were abandoned early, and new strategies tested to understand their effectiveness. An example of a non-effective strategy was adding vaccine messaging to a community gathering for a different purpose. Instead, if vaccine messaging was part of an event, the event would be advertised as a vaccine event with other attractions. This tailoring of the strategy applied to the different target groups in response to data, both quantitative and qualitative, led to vastly differing approaches to differing communities over time, despite the fact that they all started in the same place and with the same strategies. An example of this is a focus for one community on working with trusted community health services, whereas in another community, the focus was on vaccine messaging through community leaders including religious leaders. This could be compared with the build-measure-learn framework applied by the Lean Start-up model.

## Conclusion – What Can We Learn?

The South Australian COVID-19 Vaccine Hesitancy Project took place in the context of complexity and certainly required the capacity ‘to adapt both programs and the evaluation as conditions change’ (Patton, 2022). The speed and lack of detailed preparation before starting the project meant the project was driven to learn and adapt – using and privileging the available data – knowing that the project would likely not have it right from the start. This had the consequence of tailoring early to the different communities in a way that may not have occurred if the project had started off in the traditional way of extensive planning before deployment. This tension between the control and command approach where there is an orderly planned process with that plan followed through, in contrast to a complexity approach where there is uncertainty, adaptability and flexibility in the approach was recently described by Patton (2022) as one of the ten tensions in evaluation. Where uncertainty rises, it could be argued that an adaptive approach rather than a completely planned approach is warranted.

It is important to acknowledge that the Lean Start-up model in the corporate world does not guarantee success. There is a high proportion of corporate failure for a wide array of reasons (Commonwealth of Australia, 2012; Kenney et al., 2016), including where the Lean Start-up model is used (Eisenmann, 2021). For clarity, it is not suggested that adaptive approaches necessarily will lead to successful population health outcomes, but rather that there are tools and techniques that these approaches utilise that can enhance population health outcomes when used in the right circumstances and as part of a suite of actions. In particular, an explicit approach to valuing and testing early and seeking the authentic views of different community cohorts on our products in population health and adapting these to better suit in a timely way has the potential to enhance the quality of this work.

The South Australian COVID-19 Vaccine Hesitancy Project had the advantage of an extraordinary authorising environment that supported rapid collective action, but it could also be argued that this authorising environment enabled the speed but was not critical in the process of evaluative thinking. Early adaptation of products with explicit experimentation to collect actionable metrics can only improve the quality of work in population health and enhance the health and wellbeing of the population.

For the evaluation profession, this example of interest in the Lean Start-up model by population health program and policy actors indicates a need for, and interest in, adaptive approaches by our colleagues. The profession has a continuing opportunity to promote the role of evaluators in this space as complementary to formative and summative evaluation approaches. Aligned with this, this experience supports the value of ongoing promotion of evaluative thinking to program and policy colleagues who might be more familiar with the evaluation ‘doing’ role for the profession.

For government departments, as elsewhere, there is an imperative to use the available funds in the most efficient and equitable way to meet the complex challenges that face society. The model presented has demonstrated the value of adaptive approaches, particularly as uncertainty rises, and learning from models such as the Lean Start-up to improve practice and better serve the community.
